# Taxonogenomics of *Culturomica massiliensis* gen. nov., sp. nov., and *Emergencia timonensis* gen. nov., sp. nov. new bacteria isolated from human stool microbiota

**DOI:** 10.1038/s41598-023-35443-7

**Published:** 2023-05-25

**Authors:** Afaf Hamame, Reham Magdy Wasfy, Cheikh Ibrahima Lo, Florence Fenollar, Didier Raoult, Pierre-Edouard Fournier, Linda Houhamdi

**Affiliations:** 1 Aix Marseille Univ, IRD, AP-HM, MEPHI, Marseille, France; 2 IHU-Méditerranée Infection, Marseille, France; 3 Aix Marseille Univ, IRD, AP-HM, Service de Santé Des Armées (SSA), VITROME, 19-21 Boulevard Jean Moulin, 13385 Marseille Cedex 05, France

**Keywords:** Microbiology, Bacteria, Bacteriology

## Abstract

Two new bacterial strains, Marseille-P2698^T^ (CSUR P2698 = DSM 103,121) and Marseille-P2260^T^ (CSUR P2260 = DSM 101,844 = SN18), were isolated from human stools by the culturomic method. We used the taxonogenomic approach to fully describe these two new bacterial strains. The Marseille-P2698^T^ strain was a Gram-negative, motile, non-spore-forming, rod-shaped bacterium. The Marseille-P2260^T^ strain was a Gram-positive, motile, spore-forming rod-shaped bacterium. Major fatty acids found in Marseille-P2698^T^ were C_15:0 iso_ (63%), C_15:0 anteiso_ (11%), and C_17:0 3-OH iso_ (8%). Those found in Marseille-P2260^T^ strain were C_16:00_ (39%), C_18:1n9_ (16%) and C_18:1n7_ (14%). Strains Marseille-P2698^T^ and Marseille-P2260^T^ had 16S rRNA gene sequence similarities of 91.50% with *Odoribacter laneus*^T^, and of 90.98% and 95.07% with *Odoribacter splanchnicus*^T^ and *Eubacterium sulci*^T^, respectively. The exhibited digital DNA-DNA Hybridization values lower than 20.7%, and Orthologous Average Nucleotide Identity values lower than 73% compared to their closest related bacterial species *O. splanchnicus*^T^ and *E. sulci*^T^ respectively. Phenotypic, biochemical, phylogenetic, and genomic results obtained by comparative analyses provided sufficient evidence that both of the two studied strains Marseille-P2698^T^ and Marseille-P2260^T^ are two new bacterial species and new bacterial genera for which the names *Culturomica massiliensis* gen. nov., sp. nov., and *Emergencia timonensis* gen. nov., sp. nov. were proposed, respectively.

## Introduction

The human gut microbiota is considered currently as one of the most active research fields in microbiology^1^. In fact, this microflora harbours a huge biodiversity of bacteria of which a large part is still unknow^2^. Researchers used a variety of strategies to speed up and simplify the description of new bacterial species by optimizing their in vitro growth conditions^3–5^. Culturomics is one among these strategies, which relies on a diversification of culture conditions that allowed the identification of several new bacterial species isolated from the human gastrointestinal tract^3–5^. Since 2012, it allowed the isolation of over 1000 different human-associated bacterial species, including several hundreds of new species^3–6^. This method highlighted the need to adopt taxonomic approaches to clinical microbiology by including the use of modern and reproducible tools, such as high throughput genomic and proteomic analyses.

In November 30, 2015, two putative new bacterial species, *Culturomica massiliensis* gen. nov., sp. nov., and *Emergencia timonensis* gen. nov., sp. nov.*,* were isolated from patient’s stools, and partially described^7, 8^. The genomic sequencing of new described bacterial species constitutes currently a necessary step for performing their comparative taxogenomic descriptions with their closest related known species. In fact, several recent publications have used genomic descriptions to characterize the new species by comparison to their closest relatives strains^9–11^. The aim of our current study was to complete the phenotypic, taxonomic and genomic characterization proposal of new genera and new species of *Culturomica massiliensis* gen. nov., sp. nov.*,* strain Marseille-P2698^T^, and *Emergencia timonensis* gen. nov., sp. nov.*,* strain Marseille-P2260^T^ and formally expose the creation of both species.

## Materials and methods

### Sample collection and ethics approval

In November 30, 2015, stool samples were collected from hospitalized patients in the Timone Hospital (Marseille, France) as a part of a study of human microbiota diversity. Patients provided signed informed consent^7, 8^. The study protocol was approved by the ethics committee of the institut de recherche fédératif 48, under agreement number 09-022. In addition, all methods were performed in accordance with the relevant guidelines and regulations. Each sample was then cultured according to the culturomics method previously established in our laboratory^3, 5^. Various types of bacterial colonies were isolated on 5% of sheep blood-enriched Columbia agar (bioMérieux®, Marcy l’Etoile, France). Bacterial colonies were then screened for identification by Matrix-Assisted Laser Desorption Ionization-Time Of Flight Mass Spectrometry (MALDI-TOF MS) instrument (Bruker Daltonics®, Bremen, Germany) as previously reported^12^. Both two strains studied herein had a MALDI-TOF score lower than 2.0, which did not allow their correct identification. Their spectra were then added to the local MALDI-TOF MS database (https://www.mediterranee-infection.com/urms-data-base).

### 16S rRNA gene sequencing and identification

The 16S rRNA sequences from both strains were directly extracted from their whole genomes sequences and then, compared by Basic Local Alignment Search Tool nucleotide (BLASTn) to the non-redundant (nr) databases^13^. The obtained sequence similarity percentages allowed identification of the closest species to each strain, and to predict if it was new species (< 98.65% of similarity). Then, the phylogenetic tree was constructed based on these 16S rRNA gene sequences in comparison to the closest related species of each studied strain. Designated species sequences were downloaded from nr^14^, and aligned with ClustalW. Phylogenetic trees were constructed using MEGA 11 version 11.0.10 with the maximum likelihood method and 1000 bootstrap replications^15^.

### Phenotypic and biochemical characterizations

Optimal culture conditions were determined by testing various incubation temperatures (25, 28, 37, 42, and 50 °C), atmospheres (aerobic, anaerobic and microaerophilic), NaCl concentrations (5, 5.5, 7.5, 10, 15, and 20% of NaCl) and pH levels (5, 5.5, 6, 6.5, 7, 7.5, 8, 8.5). The morphology and motility were observed using a new-generation scanning electron microscope (Hitachi High-71 Technologies Corporation, Tokyo, Japan).

Furthermore, three semi-quantitative standardized micro-methods of Analytical Profile Index (API®, bioMérieux®) tests: API® 20A, API® 50 CH, and API® ZYM were used, according to the manufacturer’s instructions^16^, in order to study carbohydrate metabolism and enzymatic activities.

Fatty acid methyl ester (FAME) analysis was explored by Gas Chromatography/Mass Spectrometry, as previously reported^17, 18^. FAMEs were separated using an Elite 5-MS column and monitored by mass spectrometry (Clarus 500—SQ 8 S, Perkin Elmer®, Courtaboeuf, France). Obtained spectra were compared with those contained in the repertory databases using MS Search 2.0 operated with the Standard Reference Database 1A (National Institute of Standards and Technology-NIST, Gaithersburg, USA), and FAMEs mass spectral database (Wiley, Chichester, UK).

### Whole genomic sequencing and bioinformatic analyses

First, bacterial DNA was extracted using the EZ1 DNeasy Blood Tissue Kit (Qiagen® GmbH, Hilden, Germany) in line with the manufacturer’s protocol^19^. Whole-genome sequencing was performed using an Illumina® MiSeq sequencer (Illumina®, San Diego, CA, USA)^20^. Then, sequenced genomes were assembled using SPAdes 3.5.0 software^21^, which reduces short indels and the huge number of mismatches. Raw reads in contigs less than 700-bp-long were removed. Finally, the quality of the sequenced genome was checked using BLAST against the nr/nt database. This method allowed us to better explore the relationship between a submitted assembly of our new species to the International Nucleotide Sequence Database Collaboration (INSDC), i.e., DDBJ, ENA, or GenBank, and the assembly represented in the NCBI reference sequence (RefSeq) project. The global statistics section reported general statistics information including Gaps between scaffolds, number of scaffolds, number of contigs, total sequence length, and total ungapped length. Furthermore, taxonomic data were checked according to the best-matching-type strain with the declared new species repertory in NCBI^22^.

During annotation, genomic parameters were evaluated including transfer-messenger RNAs (tmRNAs) and transfer RNAs (tRNAs) using ARAGORN version 1.2 and ribosomal RNAs (rRNAs) using Barrnap version 0.9^23, 24^. Generated file (.*faa*) was used for BLAST-P analyses against the Clusters of Orthologous Genes (COGs) database, and used for CRISPR-Cas identification^25^. Resistance genes were screened using ResFinder^26^. Other bioinformatic tools were also used such as AntiSMASH to search polyketide synthases (PKS) and non-ribosomal peptide synthetases (NRPS)^27^. Circular maps of the two genomes were generated using CGView (Circular Genome Viewer) software. This Java application converts XML or tab-delimited input into a Vector Graphics format^28^.

Besides, phylogenetic trees of interest were generated with the FastME 2.1.6.1 software to highlight the position of each new bacterial strain among its closest relatives^29^. Digital DNA-DNA Hybridization (dDDH) values were calculated to check the difference between the genomes using the following website (https://ggdc.dsmz.de). Critical limit was set at 70% below which a prokaryotic species may be considered as new^30^. Orthologous average nucleotide identity (OrthoANI) version 0.93.1 was also used to calculate genomic similarities between studied species and their related taxa.

### Ethics approval

The study was approved by the ethics committee of the Institut de Recherche Fédératif 48 under Authorization number 09-022 with the consent of the patients.

## Results

### Strain identification and phylogenetic analyses

The species names *Culturomica massiliensis* gen. nov., sp. nov., and *Emergencia timonensis* gen. nov., sp. nov., had been previously proposed for the new species mainly as representative strains Marseille-P2698^T^ and Marseille-P2260^T^, respectively^7, 8^. As these previous descriptions were not exhaustive, we revisited the work by including phylogenetic, morphological, and genomic data.

**Figure 1:**
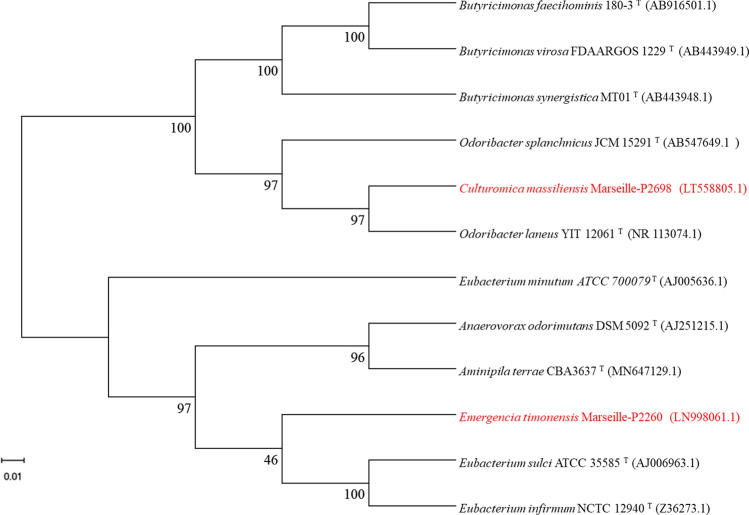
Phylogenetic tree with the position of new species (strains Marseille-P2698^T^ and Marseille-P2260^T^) among closely related species. The following phylogenetic tree was performed from the comparison of 16S rRNA sequences. The accession numbers of 16S rRNA gene are mentioned in parentheses. Bootstrap appears at the nodes. MUSCLE software was used to align sequences. The tree was designed with the MEGA-X software. The used methodology is the Maximum Likelihood method and Kimura 2-parameter model.

Strain Marseille-P2698^T^ had a 16S rRNA gene sequence similarity and a query coverage of 91.5% and 99% respectively with *Odoribacter laneus* strain YIT 12061^T^ (Fig. 1). Strain Marseille-P2260^T^ exhibited 16S rRNA gene similarity and a query coverage of 92.72%, and 100% respectively, with *Eubacterium sulci* strain ATCC 35585^T^ (Fig. 1).

### Phenotypic and biochemical characterizations

Growth of strains Marseille-P2698^T^ and Marseille-P2260^T^ occurred on 5% sheep blood-enriched Columbia agar (bioMérieux®), after 48 h of incubation at 37 °C in a strict anaerobic atmosphere. Optimal growth was obtained at pH 7. However, strain Marseille-P2260^T^ did not tolerate NaCl, whereas strain Marseille-P2698^T^ could grow with a NaCl concentration of 0.5%.

Strain Marseille-P2698^T^ is a Gram-negative rod, strictly anaerobic, motile, and non-spore-forming with a size of 1.5–3 μm in length and 0.3 to 0.4 μm in diameter. It exhibits a positive catalase, but no oxidase activity. The colonies are circular, beige and from 0.7 to 1.2 mm in diameter.

Strain Marseille-P2260^T^ is a Gram-positive, strictly anaerobic rod, ranging in length from 1 to 1.5 μm, and in diameter from 0.5 to 1 μm. It has no catalase or oxidase activity. Colonies of strain Marseille-P2260^T^ are translucent with a diameter of 0.5 to 1 mm. The remaining cell characteristics of both strains compared to their closest relatives are summarized in Table 1. Using API® 50CH strips (bioMérieux®), positive reactions were obtained for both studied strains for glycerol, d-ribose, d-galactose, d-glucose, d-fructose, d-mannose, d-mannitol, d-sorbitol, *N*-acetylglucosamine, amygdalin, arbutin, esculin ferric citrate, salicin, d-cellobiose, d-maltose, d-lactose, d-saccharose, d-trehalose, d-melezitose, gentiobiose, d-tagatose. Using API® ZYM strips (bioMérieux®), positive activities were observed for esterase lipase (C8), leucine arylamidase, phosphatase acid, and naphthol-as-bi-phosphohydrolase for both studied strains. In contrast, phosphatase alkaline, esterase (C4), α-chymotrypsin, β-galactosidase, α-glucosidase, β-glucosidase, and *N*-acetyl-β-glucosaminidase were only positive for strain Marseille-P2260^T^. Using API® 20A strips, positive results for the two strains were obtained for d-glucose, d-mannitol, d-lactose, d-saccharose, d-maltose, salicin, esculin ferric citrate, glycerol, d-cellobiose, d-mannose, d-melezitose, d-sorbitol, l-rhamnose, and d-trehalose. However, strain Marseille-P2698^T^ was positive to hydrolysis of gelatin, unlike strain Marseille-P2260^T^ which was negative.

**Table 1:** Phenotypic characteristics of strains Marseille-P2698^T^ and Marseille-P2260^T^ compared with closely related species.

Properties	*Culturomica massiliensis* gen. nov., sp. nov. Marseille-P2698^T^	*Odoribacter laneus* YIT 12061^T^	*Odoribacter splanchnicus* JCM 15291^T^	*Butyricimonas synergistica MT01*^*T*^	*Emergencia timonensis* gen. nov., sp. nov. Marseille-P2260^T^	*Eubacterium sulci* ATCC 35585^T^	*Eubacterium infirmum* NCTC 12940^T^
Gram stain	−	−	−	−	+	−	+
Cell shape	Rod	Rod	Fusiform	Rod	Rod Bacilli	Rod	Rod
Motility	+	NA	−	−	+	−	−
Cell diameter(μm)	0.7–1.2	NA	NA	1.0	0.5–1.0	1.0	1.0
Lenghth (μm)	0.3–0.4 × 1.5–3	9.2 × 1.15	0.7 × 1.0–5.0	1.5	1 to 1.5	0.5 × 1–2	0.5 × 1–2
Endospore	−	−	−	−	−	−	−
Optimum growth temperature	37 °C	37 °C	37 °C	37 °C	37 °C	37 °C	37 °C
Oxygen tolerance	Strict anaerobic	Anaerobic	Anaerobic	Anaerobic	Strict anaerobic	Anaerobic	Strict anaerobic
Incubation	4 days	4 days	2–3 days	2 days	3 days	3 days	7 days
Salt tolerance	0.5%	NA	NA	NA	−	NA	NA
Optimum pH	7	NA	NA	6–7.5	7	NA	NA
Acid phosphatase	+	+	NA	NA	+	NA	NA
Catalase	+	NA	−	−	−	NA	−
Oxidase	−	NA	NA	−	−	NA	NA
Indole	−	+	+	+	−	−	−
Urease	−	−	−	−	−	−	−
βGalactosidase	−	+	−	−	+	−	NA
Ribose	+	NA	NA	NA	+	−	−
Mannose	+	−	+	−	+	−	NA
Mannitol	+	−	NA	−	+	−	−
Sucrose	+	−	−	−	+	−	−
Glucose	+	−	+	+	+	NA	−
Fructose	+	NA	+	NA	+	NA	NA
Maltose	+	−	NA	−	+	−	−
Sorbitol	+	−	NA	−	+	−	NA
Starch	+	NA	NA	NA	−	NA	NA
Lactose	+	−	−	−	+	−	−
Source	Human faeces in diabetic patient	Human faeces	Human Abdominal abscess	Mouse feces	Human faeces	Human gingival sulcus	human periodontal pockets

+  positive result. −  negative result. *NA* not available data.

The most abundant fatty acids of strain Marseille-P2698^T^ were 13-methyl-tetradecanoic acid (63%), 12-methyl-tetradecanoic acid (11%), and 3-hydroxy-15-methyl-hexadecanoic acid (8%). Several other branched structures, mainly iso, were also detected. Specific 3-hydroxy structures were detected, mainly branched as well. For strain Marseille-P2260^T^, the major fatty acid was hexadecanoic acid (39%) (Table 2).

**Table 2:** Cellular fatty acids composition of strains Marseille-P2698^T^ and Marseille-P2260^T^.

	Fatty acids	Name	Mean relative % (a)
	15:0 iso	13-methyl-tetradecanoic acid	63.4 ± 1.8
	15:0 anteiso	12-methyl-tetradecanoic acid	10.9 ± 0.2
	17:0 3-OH iso	3-hydroxy-15-methyl-Hexadecanoic acid	8.0 ± 0.7
	16:00	Hexadecanoic acid	7.8 ± 0.3
	16:0 3-OH	3-hydroxy-Hexadecanoic acid	3.1 ± 0.3
	5:0 iso	3-methyl-Butanoic acid	1.2 ± 0.0
*Culturomica*	18:2n6	9,12-Octadecadienoic acid	1.1 ± 0.1
*massiliensis*	18:1n9	9-Octadecenoic acid	1.0 ± 0.1
Strain	15:00	Pentadecanoic acid	TR
Marseille-	14:00	Tetradecanoic acid	TR
P2698^T^	18:00	Octadecanoic acid	TR
	15:0 3-OH iso	3-hydroxy-13-methyl-Tetradecanoic acid	TR
	16:0 iso	14-methyl-Pentadecanoic acid	TR
	18:1n7	11-Octadecenoic acid	TR
	17:0 iso	15-methyl-Hexadecanoic acid	TR
	14:0 iso	12-methyl-Tridecanoic acid	TR
	16:0 3-OH iso	3-hydroxy-14-methyl-Pentadecanoic acid	TR
	17:00	Heptadecanoic acid	TR
	15:00	3-OH anteiso 3-hydroxy-12-methyl-Tetradecanoic acid	TR
	16:00	Hexadecanoic acid	39.2 ± 2.4
	18:1n9	9-Octadecenoic acid	16.5 ± 1.3
	18:1n7	11-Octadecenoic acid	14.5 ± 4.8
*Emergencia*	14:00	Tetradecanoic acid	11.9 ± 0.6
*timonensis*	18:00	Octadecanoic acid	9.5 ± 1.4
Strain	18:1n3	15-Octadecenoic acid	5.6 ± 0.9
Marseille-	18:2n6	9,12-Octadecadienoic acid	1.1 ± 0.3
P2260^T^	15:00	Pentadecanoic acid	TR
	15:0 anteiso	12-methyl-tetradecanoic acid	TR
	12:00	Dodecanoic acid	TR

a: Mean peak area percentage. *TR* trace amount.

### Genomic properties and analyses

**Figure 2:**
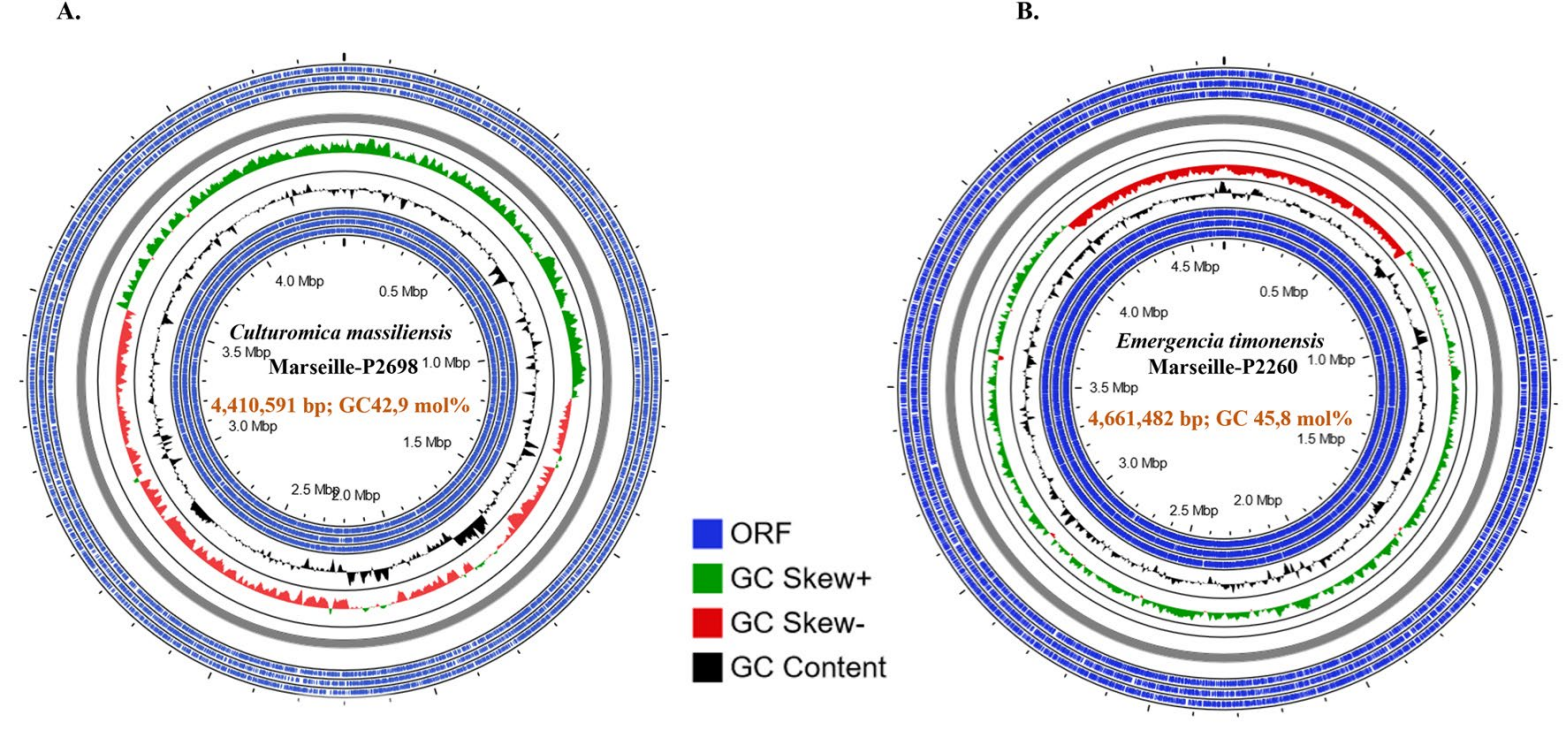
Circular genome map of strains Marseille-P2698^T^ (left) and Marseille-P2260^T^ (right) generated by the CGView software. From outside to the center: blue rings demonstrate the ORFs (Open Reading Frames) on both forward and reverse strands, green and red rings represent both positive and negative GC skew respectively, and black ring represents the GC content plot.

The whole genome of strain Marseille-P2698^T^ was composed of 14 contigs, for a total size of 4,410,591 bp, with a G+C content of 43 mol% (Fig. 2). This genome contained 3679 genes, of which 3487 were protein-coding genes. In addition, 59 RNA sequences were also identified and distributed as follows: 8 rRNAs (three 16S, two 23S, and three 5S), 51 tRNAs, and 1 tmRNA.

Genome from strain Marseille-P2260^T^ was composed of 9 contigs, with a size of 4,661,482 bp, and a 45.8 mol% G + C content (Fig. 2). Genome annotation identified 4380 genes, of which 4288 were protein-coding genes. There were 56 RNA sequences including 5 rRNAs (one 16S, one 23S, and three 5S), 51 tRNAs, and 1 tmRNA.

*Odoribacter laneus*^T^, *O. splanchnicus*^T^, *Butyricimonas synergistica*^T^*, B. faecihominis*^T^* and B. virosa*^T^ exhibited genome sizes ranging from 3.77 to 4.81 Mbp. The closest bacteria of strains Marseille-P2698^T^ and Marseille-P2260^T^ are presented in Table 3*. Eubacterium sulci*^T^*, E. infirmum*^T^*, A. terrae*^T^*, E. minutum*^T^*,* and *Aminipila odorimutans*^T^ exhibited genome sizes ranging from 1.73 to 4.66 Mbp.

**Table 3:** Summary of comparative genomes and characteristics for strains Marseille-P2698^T^ and Marseille-P2260^T^.

	Organisms	INSDC/RefSeq	Size (M bp)	G + C %	Protein-coding genes	rRNA	tRNA	tmRNA	genes	Genes assigned to COGs	Contigs	Bases	CDS
	**Strain Marseille-P2698**^T^	FLSN01000000	4.41	43	3487	8	50	1	3679	2113	11	4410591	3620
*Culturomica*	*Odoribacter laneus* YIT 12,061^T^	NZ_JH594596	3.77	40.6	2993	10	53	3	3100	2995	42	3,794,909	3095
*massiliensis*	*Odoribacter splanchnicus* JCM 15291^ T^	CP086000.1	4.39	43.4	3545	12	59	1	3757	2106	1	4,393,364	3685
strain	*Butyricimonas synergistica* MT01^T^	NR_041690.1	4.77	43.8	3741	8	54	1	3922	2113	16	4,770,838	3859
Marseille-P2698^T^	*Butyricimonas faecihominis* 180-3^ T^	NR_126194.1	4.79	42.9	3832	4	57	1	4017	2156	29	4,793,705	3955
	*Butyricimonas virosa* CCUG 56611^ T^	CP069450.1	4.81	42.4	3837	15	57	1	4032	2169	1	4,813,143	3959
	**Strain Marseille-P2260**^T^	FLKM01000000	4.66	45.8	4288	5	50	1	4380	2895	9	4,661,482	4324
*Emergencia*	*Eubacterium sulci* ATCC 35585^ T^	CP012068.1	1.73	39.9	3062	6	40	1	1646	1235	1	1,739,380	1599
*timonensis*	*Eubacterium infirmum* NCTC 12940^ T^	NZ_JH594435.1	1.91	39.9	3510	4	43	1	1781	1305	13	1,910,927	1733
strain	*Aminipila terrae* CBA3637^T^	CP047591.1	3.51	37	3102	18	65	1	3581	2346	1	3,512,404	3497
Marseille-P2260^T^	*Eubacterium minutum* ATCC 700,079 T	CP016203	1.9	45.79	1454	7	43	1	1620	1035	124	1,903,428	1246
	*Anaerovorax odorimutans* DSM_5092^T^	NZ_AUFC01000001.1	3.26	31.5	2894	4	42	1	2944	1976	54	3,263,520	2897

Strain Marseille-P2698^T^ shared dDDH values of 21.6% with *Odoribacter laneus*^T^, 20.6% with *O. splanchnicus*^T^, 30.6% with *Butyricimonas faecihominis*^T^, 21.8% with *B. virosa*^T^, and 18.9% with *B. synergistica*^T^. Strain Marseille-P2260^T^ exhibited dDDH values of 20.1% with *Eubacterium sulci*^T^, 21.1% with *E. infirmum*^T^, 23.1% with *A. terrae*^T^, 24.4% *with E. minutum*, and 21.1% with *Aminipila odorimutans* (Table 4).

**Table 4:** Comparative digital DNA-DNA Hybridization (dDDH) values (%) between studied bacterial genomes.

	CM	OL	OS	BS	BF	BV	ET	EM	AT	AO	ES	EI
CM	100%	21.6% (± 4.7%)	20.6 (± 4.7%)	18.9 (± 4.5%)	30.6 (± 4.9%)	21.8 (± 4.7%)	17.8 (± 4.4%)	16 (± 4.4%)	17.1 (± 4.4%)	21.5 (± 4.7%)	18.3 (± 4.5%)	18.2 (± 4.5%)
OL		100%	13.3 (± 6.5%)	18.6 (± 4.66%)	18.5 (± 4.5%)	19.9 (± 4.6%)	18.1 (± 4.5%)	17.7 (± 4.5%)	18.1 (± 4.5%)	21 (± 4.7%)	18.2(± 4.6%)	18.1(± 4%)
OS			100%	23.7 (± 4.8%)	19.2 (± 4.6%)	23 (± 4.8%)	19.3 (± 4.5%)	18.5 (± 4.5%)	19.1 (± 4.6%)	21.5 (± 4.7%)	19.1 (± 4.6%)	17.3 (± 4.5%)
BS				100%	21 (± 4.7%)	20.9 (± 4.7%)	19.6 (± 4.6%)	20.9(± 4.7%)	20.6 (± 4.6%)	22.9 (± 4.8%)	20.9 (± 4.7%)	18.1 (± 4.5%)
BF					100%	42.2 (± 5.1%)	43.4 (± 5.1%)	3.7 (± 2%)	3.7 (± 2%)	30.4 (± 4.9%)	3.7 (± 2%)	3.7 (± 2%)
BV						100%	18 (± 4.5%)	17.7 (± 4.5%)	17.9 (± 4.5%)	21 (± 4.7%)	18.3 (± 4.5%)	18.3 (± 4.6%)
ET							100%	24.4 (± 4.8%)	23.1 (± 4.8%)	21.1 (± 4.7%)	20.1 (± 4.6%)	21.1 (± 4.6%)
EM								100%	29.5 (± 4.9%)	31.2 (± 4.9%)	25.4 (± 4.8%)	24.9 (± 4.8%)
AT									100%	23 (± 4.8%)	28.8 (± 4.9%)	24.4 (± 4.8%)
AO										100%	19.8 (± 4.6%)	23.4 (± 4.8%)
ES											100%	28.9 (± 4.9%)
EI												100%

**Figure 3:**
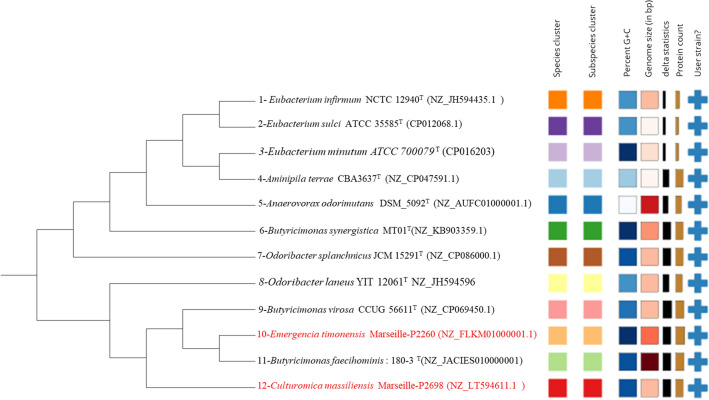
Whole-Genome sequence-based phylogenetic tree of strains Marseille-P2698^T^ and Marseille-P2260^T^ using TYGS design tree with FastME 2.1.4 software based on Genome BLAST Distance Phylogeny (GBDP) parameters. Distances were calculated from the genome sequences, and branch lengths were calculated by GBDP distance formula d5.

The phylogenetic relationships of strains Marseille-P2698^T^ and Marseille-P2260^T^ with relative strains, based on whole-genome sequencing, is represented in Fig. 3.

CM. *Culturomica massiliensi*s gen. nov., sp. nov*.*, Marseille-P2698^T^. OL. *Odoribacter laneus*^T^. OS. *Odoribacter splanchnicus*^T^. BS. *Butyricimonas synergistica*^T^. BF. *Butyricimonas faecihominis*^T^. BV. *Butyricimonas virosa*^T^. ET*. Emergencia timonensis* gen. nov., sp. nov*.*, Marseille-P2260^T^. EM*. Eubacterium minutum*^T^. AT. *Aminipila terrae*^T^. AO. *Anaerovorax odorimutans*^T^. ES. *Eubacterium sulci*^T^. EI. *Eubacterium infirmum*^T^.

**Figure 4:**
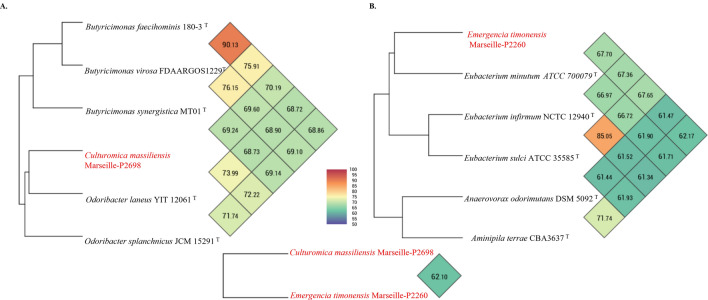
Heatmap and phylogenetic trees showing the average nucleotide identity based on calculated orthology (OrthoANI) of Marseille-P2698^T^ (**A**) and Marseille-P2260^T^ (**B**) compared to their closest related bacterial species.

The obtained dDDH values were lower than the 70% threshold used for delineating prokaryotic species^4^. In addition, strain Marseille-P2698^T^ exhibited OrthoANI values of 73.99% with *O. laneus*^T^, 72.22% with *O. splanchnicus*^T^*,* and 69.24% with *B. synergistica*^T^. Strain Marseille-P2260^T^ had an OrthoANI value of 67.65% with *E. sulci*^T^ and 67.36% with *E. infirmum*^T^. These values were lower than 95%, also suggesting that strains Marseille-P2698^T^ and Marseille-P2260^T^ belonged to distinct species (Fig. 4).

**Figure 5:**
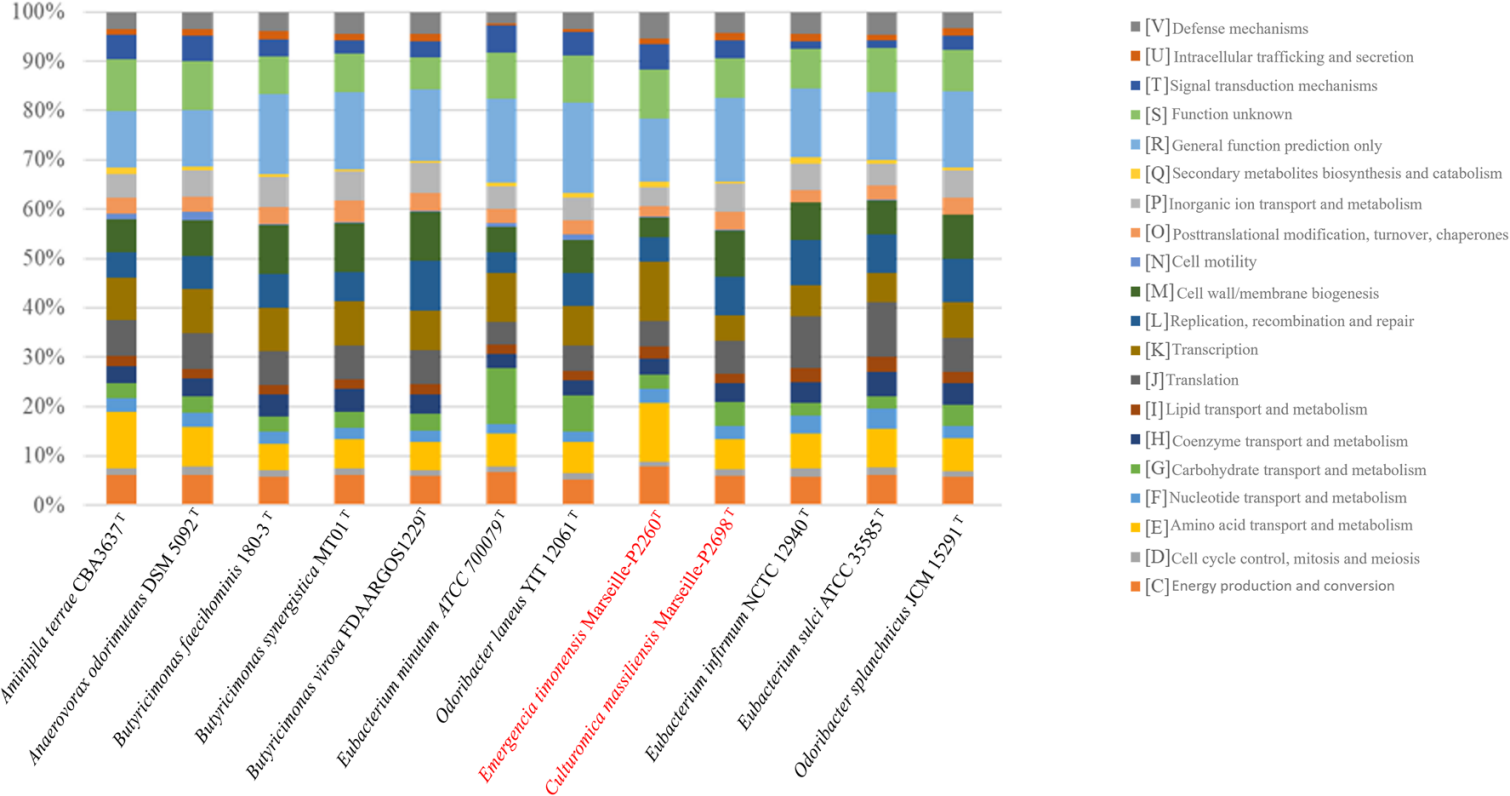
Distribution of functional classes of predicted genes expressing group of proteins that clusters according to functions.

Genes encoding proteins are divided into several categories according to their functions and others have unknown functions. COGs of strains Marseille-P2698^T^ and Marseille-P2260^T^ have different functional distributions from their closest species (Fig. 5).

### Antibiotic resistance genes and defense mechanisms

Using the ResFinder software, antibiotic resistance genes (ARG) *erm* (F) and *tet* (Q) were detected within the genome of strain Marseille-P2698^T^, with identity percentages of 100% and 99.8%, respectively. For strain Marseille-P2260^T^, ARG included *erm* (B), *tet* (M), and *tet* (O), with identity percentage ranging from 99.7% to 100% (Table 5).

**Table 5:** Antibiotic resistance genes detected in the genomes strains Marseille-P2698^T^ and Marseille-P2260^T^ using ResFinder software.

	Resistance gene	Identity	Alignment Length/Gene Length	Position in contig	Phenotype
	**Macrolide**				
	erm(F)	100	801/801	1,826,565.0.1827365	erythromycin,lincomycin,clindamycin,quinupristin,pristinamycin ia,virginiamycin s
	**Lincosamide**				
*Culturomica*	erm(F)	100	801/801	1,826,565.0.1827365	erythromycin,lincomycin,clindamycin,quinupristin,pristinamycin ia,virginiamycin s
*massiliensis*	**Streptogramin b**				
Marseille-P2698^T^	erm(F)	100	801/801	1,826,565.0.1827365	erythromycin,lincomycin,clindamycin,quinupristin,pristinamycin ia,virginiamycin s
	**Tetracycline**				
	tet(Q)	99.8	1926/1926	1,055,038.0.1056963	doxycycline,tetracycline,minocycline
	**Macrolide**				
	erm(B)	100	738/738	1,359,368.0.1360105	erythromycin,lincomycin,clindamycin,quinupristin,pristinamycin ia,virginiamycin s
*Emergencia*	**Lincosamide**				
*timonensis*	erm(B)	99.86	738/738	1,359,368.0.1360105	erythromycin,lincomycin,clindamycin,quinupristin,pristinamycin ia,virginiamycin s
Marseille-P2260^T^	**Streptogramin b**				
	erm(B)	99.9	738/738	1,359,368.0.1360105	erythromycin,lincomycin,clindamycin,quinupristin,pristinamycin ia,virginiamycin s
	**Tetracycline**				
	tet(M)	99.84	1920/1920	1,222,665.0.1224584	doxycycline,tetracycline,minocycline
	tet(O)	99.79	1920/1920	2,008,154.0.2010073	doxycycline,tetracycline,minocycline

The genomes of strains Marseille-P2698^T^ and Marseille-P2260^T^ contained CRISPR-Cas modules (Table 6). Strain Marseille-P2698^T^ contains one defense mechanism composed of polyketide synthases (PKS) and non-ribosomal peptide synthetases (NRPS) enzymes. NRPS-PKS was previously demonstrated to have a role in the biosynthesis of pharmaceutically-important natural products^31^. Using ANTI SMASH software, NRPS-PKS-like genes cluster had been detected in genome of strain Marseille-P2698^T^, and β-lactone; containing potent medicinal properties^32, 33^, had been detected in strain Marseille-P2698^T^ genome. In addition, according to our results in ToxFinder-1.0 software detecting toxins^34^, our two strains did not possess toxin genes.

**Table 6:** Identification of CRISPR-Cas type and subtype genes in user-submitted sequence for strains Marseille-P2698^T^ and Marseille-P2260^T^.

Bacteria	Element	CRISPR Id /gene name	Start	End	Spacer /Gene	Repeat consensus /cas genes	Orientation
	CRISPR		112	199	1	ATGGGACTCTTTTTTTGTAAATA	
	Cas cluster	CAS	71,544	73,337	1	Cas3_0_I	+
	Cas cluster	CAS-TypeIB	613,607	622,650	7	Cas6_0_I-III, Cas7_3_IB, Cas5_1_IB, Cas3_0_I,	+
		Cas6_0_I-III				Cas4_0_I-II, Cas1_0_I-II-III, Cas2_0_I-II-III-V	
*Culturomica*	CRISPR	Cas7_3_IB	622,870	625,330	37	CTTTTAATTGAACTAAGGTAGAATTGAAAC	+
*massiliensis*	CRISPR	Cas5_1_IB	992,376	993,764	21	GTTTCAATACTACTTAGTTCTATTAAAAG	+
Marseille-P2698^T^	CRISPR	Cas3_0_I	2,048,339	2,049,405	15	GTCTCAATGCCGTGATTACAGCTATTCTCTAAATC	+
	Cas cluster	Cas4_0_I-II	2,049,852	2,057,279	6	Cas2_0_I-II-III-V, Cas1_0_I-II-III, Cmr6_0_IIIB,	+
						Cmr4_0_IIIB,Cmr3_0_IIIB, Cas10_1_IIIB	
	CRISPR	Cas1_0_I-II-III	3,461,867	3,462,226	5	ATTTCAATTCTACTCCAGTTCTATTAAAAT	+
	CRISPR	Cas2_0_I-II-III-V	3,750,732	3,750,812	1	AATTCTCTTTAGCGTTGGTTTGTG	+
	CRISPR		882	1086	2	GGCATATATTATCAATACAGTATTTCCCTATTTT	
	CRISPR		300,401	300,525	1	TTGCGGCAACGCCGTGAGATTTTGGAATCATTATA	+
	CRISPR		522,369	522,503	1	TTGCAGCAACGCTGTCAATATCGTTGTGTTCATTATACCATGA	+
	CRISPR		1,380,564	1,381,183	9	GTCGCATCCTCATGGATGCGTGGATTGAAAT	−
	CRISPR	CAS-TypeIC	1,429,920	1,430,223	4	GTCTTGCTCCGCATGGAGCAAGTGGATTGAAAT	+
	Cas cluster		1,453,194	1,461,082	7	Cas3_0_I, Cas5_0_IC, Cas8c_0_IC, Cas7_0_IC, Cas4_0_I-II, Cas1_0_IC, Cas2_0_I-II-III-V	
*Emergencia*	CRISPR		1,461,248	1,463,328	30	GTCGCACTCCGCATGGAGTGCGTGGATTGAAAT	+
*timonensis*	CRISPR		1,608,002	1,608,146	1	AAAGCATAAGGACTTTCATCAATTTGTTTCACAAATTGTGACAAGTCCATTA	-
Marseille-P2260^T^	CRISPR		1,944,682	1,944,814	1	GGTATAATGAATCCATAACCAAACGACGTTGTCGCAAGGTAG	+
	CRISPR		225,209	225,286	1	ATCCAAGCCAATTTCTTATCGAGGCT	−
	CRISPR		530,585	530,712	1	TATTTTCAGCGGAATGCACATGGTATAATAAACGCA	−
	CRISPR		853,819	853,957	1	TGGTATAATGGTTCCACGATCTTACGGCGTTGCCGAAGTGTAGGAACCA	+
	CRISPR		1,673,013	1,673,146	1	CATTATACGACAAAACGCAGCCAAATCAGAGATTTGGGCGTT	+
	CRISPR		1,798,696	1,798,841	1	TCCTTATGCTTTAAACGCCTAAATCTGTGATTTAGCTGCGTTTTGTCGTATAA	−
	CRISPR		1,910,685	1,910,831	1	AATGAACGCTTCCTTGTCAGCAATCTTGATTAAGATTGTGCGTTTATTATACCA	−
	CRISPR		1,927,335	1,927,459	1	CCGTTCATCTTTCGGCGTTGCCGCAAGGTAGGAA	+
	CRISPR		2,025,969	2,026,111	1	TGTTCTGCTTAACTGACTCCATATCCAGCCCTCTGTGTTACGCAGATTT	+

## Conclusion

Culturomics combined with Taxogenomics allowed the isolation and the full characterization of two new bacterial species isolated from human intestine. Primitive phylogenetic comparisons were previously performed^7, 8^, while a thorough phylogenetic and genomic analyses were performed in our study. The results of phenotypic, biochemical, phylogenetic, and genomic analyses obtained for both studied strains proved that they belong to new species. Thus, we propose the creation of two new bacterial genera and new species, *Culturomica massiliensis* gen. nov., sp. nov., and *Emergencia timonensis* gen. nov., sp. nov..

### Description of *Culturomica* gen. nov

*Culturomica* (*Cul.tu.ro.mi’ca*. N.L. fem, *culturomica*, referring to a new method of diversified bacterial culture). Cells are anaerobic, Gram-negative, motile and rod-shaped. The type species *is Culturomica massiliensis* gen. nov., sp. nov**.**. It was isolated from human feces.

### Description of *Culturomica massiliensis* gen. nov., sp. nov

*Culturomica massiliensis* (*mas.si.li.en’sis*. L. fem. adj.*massiliensis*, form “*Massilia*”, the Latin name of Marseille, France, where the type strain was iso-lated). Bacterial cells are Gram-negative and rod-shaped. They are motile and non-spore-forming, with lengths ranging from 1.5 to 3.0 µm, and diameters from 0.3 to 0.4 µm. The optimal growth conditions are 37 °C, a strict anaerobic at-mosphere, and neutral pH. On 5% sheep blood-enriched Columbia agar, colonies appear small, circular, beige to white, and measure between 0.7 and 1.2 mm in diameter. They ferment d-galactose, d-glucose, d-fructose, d-mannose, inosi-tol, d-mannitol, d-sorbitol, *N*-acetylglucosamine, amygdalin, arbutin, esculin, salicin, d-cellobiose, d-maltose, d-glucose, glycerol, d-lactose, d-saccharose, d-trehalose, d-melezitose, Amidon, gentiobiose, d-tagatose, potassium gluconate and potassium 5-ketogluconate. Enzymatic activities for phosphatase al-kaline, esterase, esterase lipase, leucine arylamidase, α-chymotrypsin, phospha-tase acid, naphthol-as-bi-phosphohydrolase, β-galactosidase, α-glucosidase, β-glucosidase, and *N*-acetyl-β-glucosaminidase are present. The major cell wall fatty acids are C_15:0 iso_ (63%), C_15:0 anteiso_ (11%), and C_17:0 3-OH iso_ (8%). The genome size from strain Marseille-P2698^T^ is 4.41Mbp-long with a G+C content of 43 mol%.

The type strain Marseille-P2698^T^ (CSUR P2698 = DSM 103,121) was isolated from a stool specimen of a 66-year-old man with diabetes mellitus.

The accession numbers for the genomic and 16S rRNA gene sequences of strain Marseille- P2698^T^ are deposited in the GenBank database under references FLSN00000000 and LT558805, respectively.

### Description of *Emergencia* gen. nov

*Emergencia timonensis* (*e.mer.gen’cia* N.L. fem. n., *Emergencia,* for emergence, in reference to the discovery of emerging human bacteria).

### Description of *Emergencia timonensis* gen. nov., sp. nov

*Emergencia timonensis* (ti.mo.nen’sis. L. fem. adj., timonensis, from Timone, the name of a university hospital in Marseille, France, where the type strain was isolated).

Bacterial cells are Gram-positive rod-shaped and bacilli. They are motile, non-spore-forming, with lengths ranging from 1.0 to 1.5 µm, and a diameter of 0.5 to 1.0 µm. The optimal growth conditions are 37 °C, a strict anaerobic atmosphere, and pH.7. On 5% sheep, blood-enriched Columbia agar, colonies of strain Marseille-P2260^T^ appear small, translucent, and measure from 0.7 to 1.2 mm in diameter. Cells ferment d-glucose, d-mannitol, d-lactose, d-saccharose, d-maltose, salicin, esculin ferric citrate, glycerol, d-cellobiose, d-mannose, d-melezitose, d-sorbitol, l-rhamnose, d-trehalose, glycerol, d-arabinose, l-arabinose, d-ribose, d-xylose, d-galactose, d-glucose, d-fructose, d-mannose, l-rhamnose, dulcitol, d-mannitol, d-sorbitol, Methyl-αd-glucopyranoside, *N*-acetylglucosamine, amygdalin, arbutin, esculin, salicin, d-cellobiose, d-maltose, d-lactose, d-melibiose, d-saccharose, d-trehalose, d-melezitose, d-raffinose, gentiobiose, d-turanose, d-tagalose, l-fucose, and potassium gluconate. In addition, enzymatic activities such as esterase lipase, leucine arylamidase, acid phosphatase, and naphthol phosphohydrolase are present. The major cell wall fatty acids are C_16:00_ (39%), C_18:1n9_ (16%), and C_18:1n7_ (14%).

The genome size from strain Marseille-P2260^T^ is 4.66 Mbp with a G+C content of 45.8 mol%.

The type strain Marseille-P2260^T^ (CSUR P2260 = DSM 101,844 = SN18) was isolated from a stool sample the feces of a healthy patient with an unremarkable medical history.

## Data Availability

The accession numbers for the genomic and 16S rRNA gene sequences are deposited in the GenBank database under references: FLSN00000000 and LT558805 respectively for Marseille- P2698^T^ strain, and FLKM00000000 and LN998061 respectively for Marseille- P2260^T^ strain.
